# Editorial: Treatment response and resistance to targeted therapies for NSCLC

**DOI:** 10.3389/fonc.2026.1780209

**Published:** 2026-03-26

**Authors:** Shiyou Wei, Zhi Wei, Yu Liu, Yongchang Zhang, Gao Zhang

**Affiliations:** 1 Department of Thoracic Surgery, West China Hospital, Sichuan University, Chengdu, Sichuan, China; 2 Department of Computer Science, Ying Wu College of Computing, New Jersey Institute of Technology, Newark, NJ, United States; 3 Department of Biology and Biochemistry, University of Houston, Houston, TX, United States; 4 Department of Medical Oncology, Lung Cancer and Gastrointestinal Unit, Hunan Cancer Hospital and The Affiliated Cancer Hospital of Xiangya School of Medicine, Central South University, Changsha, China; 5 Faculty of Dentistry, the University of Hong Kong, Hong Kong, Hong Kong SAR, China

**Keywords:** editorial, non-small cell lung cancer, targeted therapies, treatment resistance, treatment response

Targeted therapies have revolutionized the treatment of non-small cell lung cancer (NSCLC), offering a greater potency and fewer side effects compared to conventional radiotherapy and chemotherapy (1, 2). Despite their dramatic initial efficacy, acquired resistance almost invariably develops within one to two years after treatment initiation (3–5). Molecular mechanisms underlying treatment response and resistance in patients with NSCLC are still not fully elucidated. This Research Topic, entitled “Treatment Response and Resistance to Targeted Therapies for NSCLC,” brings together 16 diverse contributions, including original research articles, case reports, and reviews, providing a timely overview of recent advances in the field.

Several articles systematically reviewed the current landscape and mechanisms of resistance to targeted therapies. Yu et al. comprehensively summarized the mechanisms and present status of targeted therapy for NSCLC, discussing strategies to overcome acquired resistance and ongoing challenges. Similarly, Zhang et al. outlined targeted therapies for NSCLC and offer insights into treatment response and mechanisms of resistance. Focusing on a specific pathway of resistance, Jiang et al. summarized progress in understanding bypass activation mechanisms related to resistance to tyrosine kinase inhibitor.

The complexity of resistance was further illustrated through reports of specific genetic alterations and clinical cases. Zeng et al. described two cases of resistance to EGFR-TKIs mediated by activation of the ALK and BRAF bypass signaling pathways. Guo et al. reported a patient who acquired *EML4-ALK* fusion after sequential treatment with erlotinib and osimertinib and subsequently achieved a durable response to ensartinib. Wang et al. presented two patients with NSCLC harboring *EGFR* fusion mutations combined with *EGFR* amplification who exhibited divergent responses to almonertinib, highlighting the complexity introduced by co-occurring molecular abnormalities. Delving into structural specificity, Guan et al. investigated how the *ALK^F1174S^* mutation influences kinase activity and drug sensitivity in *EML4-ALK* variants 1 and 3, underscoring the intricacy of drug selection in the presence of mutations conferring resistance.

Research on therapies targeting specific oncogenic drivers also provides new evidence and perspectives into the area of targeted therapies. Seong et al. reported a case of *ALK*-rearranged NSCLC achieving pathological complete response following neoadjuvant brigatinib. Niu et al. described a patient with *ALK*-positive NSCLC and a paraneoplastic leukemoid reaction who responded to fourth-line lorlatinib. Nishiyama et al. presented a case of *BRAF^K601E^*-mutant NSCLC with limited effectiveness of dabrafenib and trametinib, pointing to the persistent challenges in treating *BRAF*-mutant NSCLC. Meng et al. demonstrated the therapeutic activity of tepotinib in four patients with NSCLC harboring *MET* exon 14 skipping mutations.

Further studies explored combination strategies, novel targets, and adverse events. Jin et al. examined first-line TKI efficacy in *PD-L1*-positive, *EGFR*-mutated NSCLC and find that adding antiangiogenic agents and immunotherapy can significantly improve survival outcomes. Pan et al. reported long-term survival in a patient with *EGFR*-mutated/*HER2*-amplified NSCLC treated with a combination of almonertinib and pyrotinib. Oduah et al. explored the potential efficacy of proteasome inhibitors in *TP53*-mutated NSCLC. Sentek et al. investigated the role of phenotype transition toward tumor-associated mesenchymal stem cells in resistance of NSCLC to treatment. Finally, Wheatley-Price et al. reported two cases of appendicitis as a rare potential adverse event associated with alectinib treatment.

In summary, targeted therapy for NSCLC continues to face significant challenges. Acquired resistance remains a major barrier, driven by diverse and complex mechanisms including bypass signaling, phenotypic transformation, and compound mutations. Moreover, the heterogeneous molecular background of tumors leads to markedly varied patient responses even to the same targeted agent. Moving forward, a deeper understanding of the biology of resistance, more precise molecular screening, stratification and dynamic monitoring, and the development of novel mechanism-based combination strategies will be essential to improve long-term outcomes for patients.
